# EZ-Pair graph: scalable unified-axis visualization method for summarizing large-scale paired data

**DOI:** 10.1093/bioadv/vbag155

**Published:** 2026-06-02

**Authors:** Akihiro Ezoe, Motoaki Seki, Keiichi Mochida

**Affiliations:** RIKEN Center for Sustainable Resource Science, Yokohama, Kanagawa 230-0045, Japan; RIKEN Center for Sustainable Resource Science, Yokohama, Kanagawa 230-0045, Japan; RIKEN Center for Sustainable Resource Science, Yokohama, Kanagawa 230-0045, Japan

## Abstract

**Motivation:**

Avoiding dual-axis visualization improves quantitative interpretation. Yet for large-scale paired datasets, combining raw data with summary metrics on dual axes often hinders interpretability, while single-axis displays risk visual saturation of paired lines. These limitations may be overcome by developing summary metrics that can be plotted on the same axis as the underlying data.

**Results:**

We developed EZ-Pair Graph as a suite of highly scalable methods that aggregate positional and slope information of numerous lines into a unified and interpretable axis. EZ-Pair Graph comprises three complementary tools: trapezoid plot, which summarizes ascending and descending groups of paired differences and their prevalence, and the clustered line or parallel arrow plot, which can reveal clustered patterns and directional heterogeneity in paired differences. By selectively emphasizing the rank and magnitude of paired differences, these plots facilitate the interpretation of distributional differences in large-scale paired data. We demonstrate the effectiveness of our methods using biological datasets that are difficult to visualize using conventional approaches. In each case, our methods revealed structured, localized, and heterogeneous trends through clear visual summaries. As datasets increase in scale and complexity, EZ-Pair Graph may be useful for detecting underlying patterns and localized variations that are often overlooked in conventional paired-data visualizations.

**Availability and implementation:**

https://github.com/010049nn/EZ_pair_graph; EZ-Pair Graph outputs are available in multiple formats (PDF, SVG, PNG, HTML, and JSON). Installation via pip and docker is possible. Release archive DOI: 10.5281/zenodo.20437542.

## 1 Introduction

Positioning data along a common scale substantially enhances interpretability (Cleveland and McGill 1985). However, displaying numerous paired observations on a single axis rapidly leads to visual saturation, as overlapping lines become indistinguishable even at moderate sample sizes—a well-characterized challenge in information visualization known as overplotting or visual clutter (Ellis and Dix 2007, Mayorga and Gleicher 2013). A natural remedy is to introduce summary statistics, yet conventional summary metrics—such as means, confidence intervals, or effect sizes—typically require a separate axis or annotation layer, reintroducing the dual-axis problem that compromises quantitative comparison (Liu *et al.* 2014). This creates a fundamental dilemma: preserving a unified axis sacrifices scalability, while achieving scalability through summarization sacrifices axis unity. Developing a visualization method that resolves this tension—scalably summarizing paired data while maintaining a single interpretive axis—would therefore represent a substantial advance in data interpretability.

**Figure 1:**
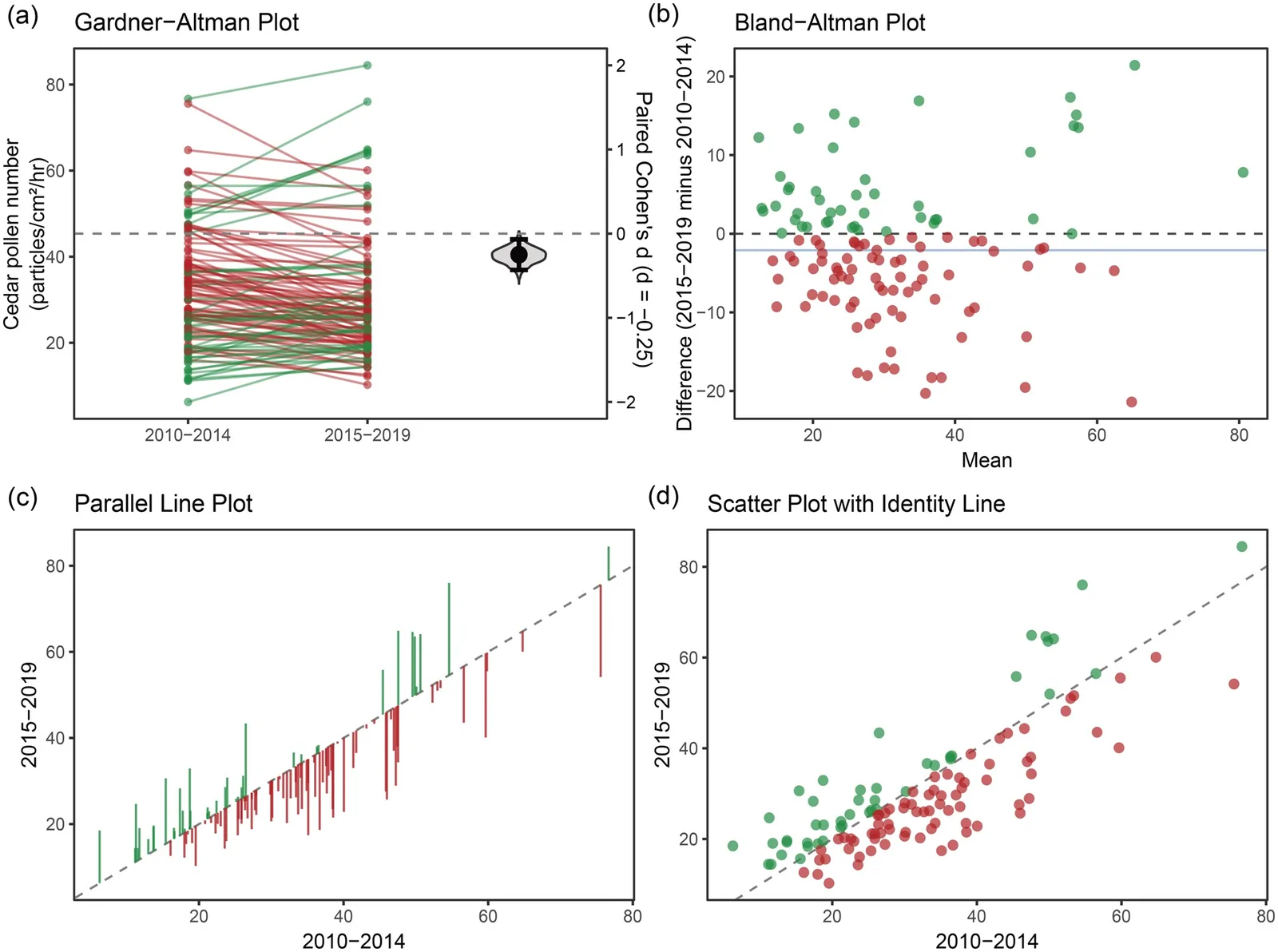
Conventional visualization methods using cedar pollen data between 2010–2014 and 2015–2019 (*n = *120 locations). (a) Gardner-Altman plot showing paired data with Cohen’s *d* = −0.25 (95% CI: −0.43 to −0.07); left and right axes present the pollen count (particles/cm^2^/h) and Cohen’s *d*, respectively. (b) Bland-Altman plot; blue line indicates the mean difference. (c) Parallel line plot sorted by 2010–2014 values; vertical lines connect paired observations. (d) Scatter plot with identity line; dashed line represents no change. Data points above and below the dashed line indicate increases and decreases in the pollen count, respectively.

For a comparison with established approaches for visualizing paired data, we generated plots using a dataset with a moderate sample size (*n = *120). Among these approaches, the Bland-Altman plot (difference plot, MA plot for genomics), which displays differences as a scatter plot, and the paired data option of the Gardner-Altman plot (estimation plot), which displays standardized effect sizes, are widely employed (Fig. 1a and b) (Ho *et al.* 2019, Wilcox and Rousselet 2023, Mazdziarz *et al.* 2025). However, the difference plot is a scatter-based representation that lacks scalability. The Gardner-Altman plot achieves scalability by summarizing distributional differences using Cohen’s *d*, but condenses the information into a single metric displayed on a separate axis. For example, Cohen’s *d* indicates the predominant direction (ascending or descending) in the data driving a statistical result, but it obscures the actual proportions and magnitudes of each group. Such information is essential for understanding the distribution of the differences underlying paired statistical tests (Rousselet *et al.* 2017). Alternative approaches, including the parallel line plot and the hybrid parallel line plot, have been proposed to display differences on a unified axis; however, scalability is not achievable with these methods (Fig. 1c) (Schriger 2018). Other approaches, such as the colored line plot and the scatter plot with identity line (Fig. 1d), extend scalability to several hundred data points, but remain incapable of handling thousands of data points without visual saturation (Nagarajan 2020). Although common methods, such as alpha blending (transparency) or constructing two-dimensional density plots with color coding, can mitigate overplotting in scatter-based representations, these approaches do not provide explicit summary metrics on the same single axis as the raw data nor can they depict differences as intuitive slope lines. Thus, despite the unprecedented ease of acquiring large-scale datasets, visualization methods that both display paired differences on a unified axis and scale to massive datasets remain scarce.

In this study, we developed the EZ-Pair Graph framework, which aggregates positional and slope information from numerous paired connections in large-scale paired datasets into a concise and interpretable visual summary, while preserving the directional and magnitude information critical for interpretation. EZ-Pair Graph comprises the following three tools: trapezoid plot, clustered line plot, and parallel arrow plot. This framework is particularly powerful for datasets characterized by small effect sizes, but substantial real-world impact (e.g. population dynamics or chronic disease interventions), where large samples are required to demonstrate that many individuals share a common trend despite modest individual changes. Thus, using EZ-Pair Graph represents a new approach to revealing subtle but consistent differences that have long been a challenge for effective visualization.

## 2 Methods

### 2.1 Overall workflow

The fundamental challenge lies in summarizing data and preserving both positional and directional information of connection lines on a single interpretable scale; this information is critical for accurate statistical interpretation. Our approach addresses this challenge through a three-stage process: (i) grouping connection lines by directional patterns to prevent the cancelation of opposing trends during summarization, (ii) sorting paired differences within each group, and (iii) applying summary statistics. This workflow ensures that visualizations faithfully reflect underlying data trends. This method is compatible with diverse clustering strategies and summary statistics. EZ-Pair Graph is available as a Python package (pip install git+https://github.com/010049nn/EZ_pair_graph.git) with both a Python API and a command-line interface. It supports multiple output formats: PDF (editable vector, default), SVG, PNG, HTML, and JSON. Docker is also available.

### 2.2 Dataset preparation

We retrieved census population data for all 1896 municipalities in Japan for 2010 and 2015 from an online source (https://ktgis.net/mandara/). We also obtained pollen count data from 120 monitoring locations across Japan for 2010–2019 from the Ministry of the Environment (https://www.env.go.jp/page_00209.html). To construct paired samples, we calculated the average pollen count (particles/cm^2^/h) from February to May (i.e. primary pollen season in Japan) at each monitoring site to serve as the representative annual value. Expression patterns of the corresponding gene pairs were obtained from a previous study (Ramírez-González *et al.* 2018). These datasets were used as practical examples for analysis.

### 2.3 Procedure for the trapezoid plot

Paired data differences were visualized as trapezoids, with positive (ascending) and negative (descending) groups displayed separately.

Procedure (Fig. 2):

**Figure 2:**
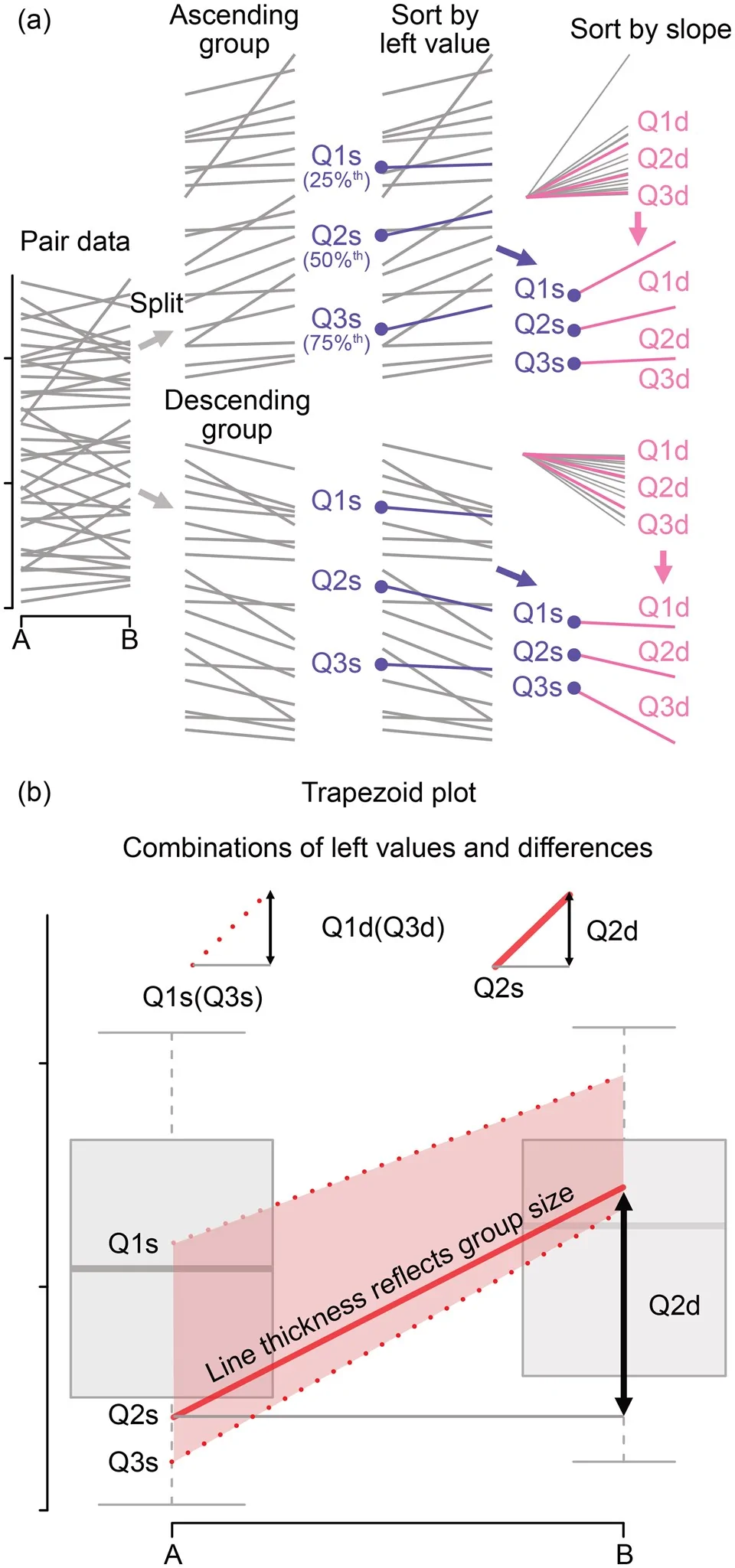
Trapezoid plot construction. (a) Paired data are split into ascending (positive differences) and descending (negative differences) groups. For each group, quartiles (25th, 50th, and 75th percentiles) are calculated for group A values (Q1s, Q2s, and Q3s) and paired differences (Q1d, Q2d, and Q3d). Quartile points and the corresponding slopes are connected to form a summary representation. (b) Trapezoid plot illustrating combinations of starting points and paired differences. Note that the pattern of steep slopes at extreme quartiles may be due to bivariate noise alone, and do not necessarily indicate a biologically meaningful trend.

1. Calculate paired differences between groups A and B as B*i* − A*i*, where *i* = 1…*n*.
2. Split the paired differences into ascending (positive), descending (negative), and identical groups. Identical values are excluded from further analysis (Fig. 2a).
3. Sort the ascending and descending groups by their corresponding group A values in descending order. Define Q1s, Q2s, and Q3s as the 25th, 50th, and 75th percentiles of these group A values, respectively (Fig. 2a).
4. For each of the ascending and descending groups, the 25th, 50th, and 75th percentiles for the differences are referred to as Q1d, Q2d, and Q3d, respectively (Fig. 2a).
5. Draw summary lines using the positions and slopes defined by Q1s–Q1d and Q3s–Q3d (Fig. 2a and b).
6. Scale the thickness or color of the Q2 line to be proportional to the number of elements in the ascending and descending groups (Fig. 2b).

### 2.4 Clustering method

Clustering was performed using hierarchical clustering or HDBSCAN to ensure reproducibility (Campello *et al.* 2013). By default, agglomerative hierarchical clustering using Ward’s minimum variance method was applied, with the optimal number of clusters determined automatically using the elbow method based on the within-cluster sum of squares (Trianasari 2024). Both hierarchical clustering and HDBSCAN produce deterministic results, thereby avoiding the initialization effects inherent to algorithms such as *k*-means (Liao *et al.* 2024). Before clustering, observations were grouped by the direction of change (B − A ≥ 0: ascending or B − A < 0: descending), and clustering was performed independently within each group. Within each group, each observation was represented as a two-dimensional point (A, B), and clusters were identified according to Euclidean distance in this coordinate space. Clusters falling outside the 1.5 × IQR whisker range were treated as outliers and excluded from visualization.

### 2.5 Clustered line plot and parallel arrow plot

To mitigate visual saturation when visualizing large-scale paired data, we introduced two clustering-based methods (clustered line plot and parallel arrow plot) that build upon the clustering procedure described in Section 2.4. Unlike the trapezoid plot, which collapses data into only two groups (ascending and descending), these plots summarize each cluster separately, thereby presenting the underlying data distribution more intuitively.

The clustered line plot extends the Q2 summary line used in the trapezoid plot by drawing one such line per cluster: Q2s for ascending clusters and Q2d for descending clusters. Color intensity encodes cluster size. The parallel arrow plot renders these summary lines as parallel arrows positioned between the two boxplots. For each cluster, the slope line is treated as the hypotenuse of a right triangle, and its vertical (opposite) component is drawn as the vertical segment of the arrow. Accordingly, the vertical portion of the arrow directly represents the magnitude of change.

## 3 Results

### 3.1 Trapezoid plot

To demonstrate how the trapezoid plot appears under different data conditions, we developed four scenarios that combined two sample sizes (large, *n = *1000; moderate, *n = *300) with two effect magnitudes: one in which paired differences are large and consistent and another in which they are small and variable. These scenarios were designed to illustrate plot behavior across a range of realistic conditions rather than to test a specific statistical hypothesis (Fig. 1, Datasets 1–4, available as supplementary data at *Bioinformatics Advances* online). Using this method, paired differences were clearly summarized and displayed on a common axis (Fig. 1, available as supplementary data at *Bioinformatics Advances* online). By avoiding slope saturation, this approach scales effectively to large datasets. A limitation of this approach is that multimodal or skewed distributions are represented by a single summarized line, potentially obscuring a clear distributional structure. This limitation was detailed in the following Section 3.3.

### 3.2 Clustered line plot and parallel arrow plot

**Figure 3:**
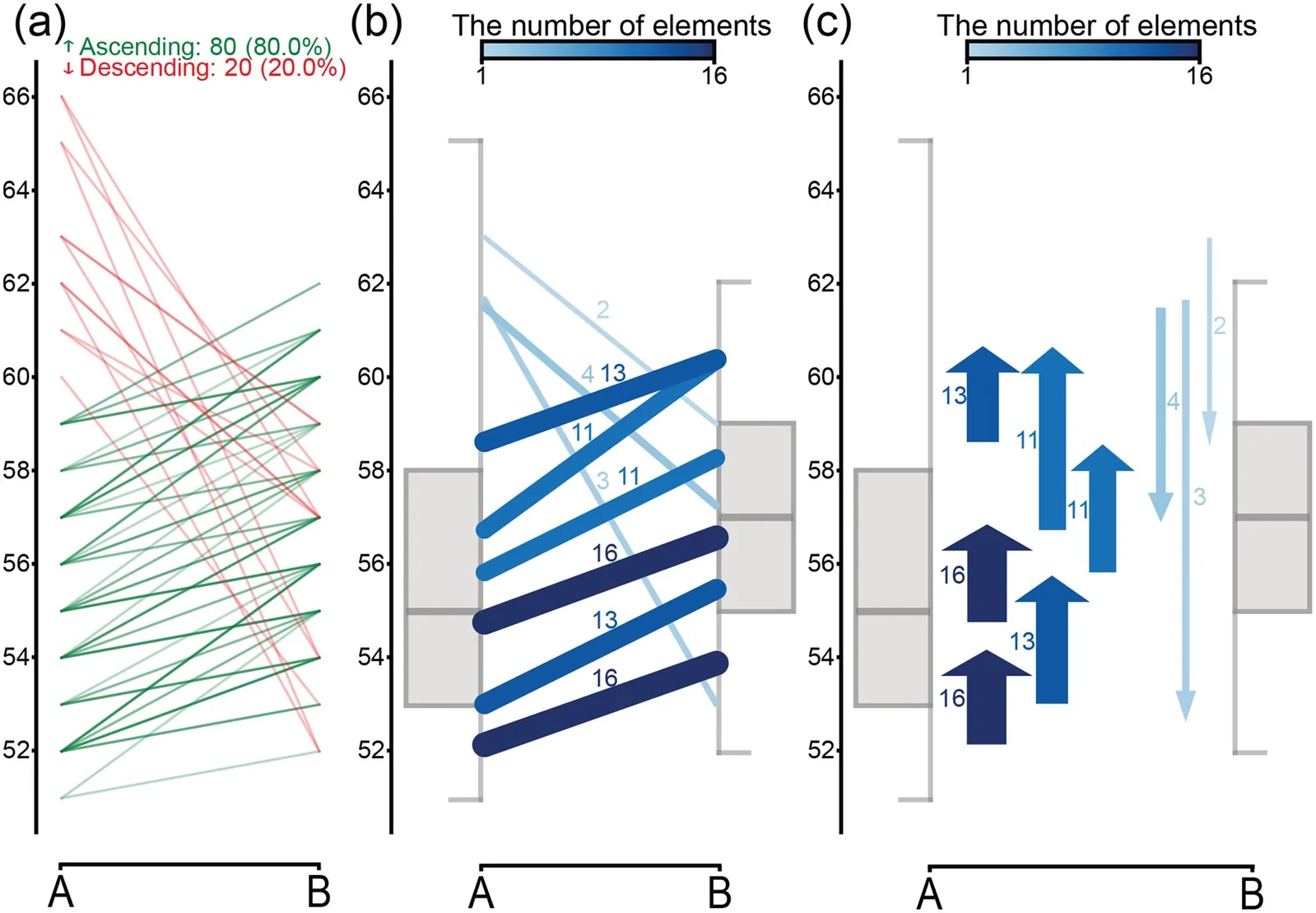
Clustered line plot and parallel arrow plot. (a) Slope graph showing extensive line overlapping (Dataset 5, available as supplementary data at *Bioinformatics Advances* online). (b) Lines clustered into nine groups and represented by Q2s/Q2d summary lines. Color and thickness indicate cluster size. (c) Parallel arrow plot. The slope line of each cluster represents the hypotenuse of a right triangle, and the opposite side (vertical component) is presented as a vertical line segment of an arrow. Upward arrows (left) indicate ascending groups and downward arrows (right) indicate descending groups. Arrow thickness and color reflect group size.

We assessed the utility of this method using Dataset 5, available as supplementary data at *Bioinformatics Advances* online (100 paired samples) because the conventional method for data visualization results in extensive line overlapping (Fig. 3a). The algorithm first separated paired connection lines into ascending and descending groups and applied hierarchical clustering within each group (Fig. 3b). Each cluster was represented by its median line (Q2s and Q2d), with color intensity indicating cluster size. In the parallel arrow plot, these summary lines were presented as parallel arrows, with vertical segments between boxplots representing differences. For each cluster, the slope line was considered as the hypotenuse of a right triangle, and the opposite side (vertical component) was presented as a vertical line segment of an arrow (Fig. 3c).

### 3.3 Visual patterns of sample size, true differences versus regression to the mean, subgroup heterogeneity, skewed distributions, and robustness to heteroscedasticity and outliers

Visual patterns under different data conditions were illustrated, with the following four scenarios: large (*n = *1000) and moderate (*n = *300) sample sizes, each with and without statistically significant paired differences (Fig. 1, Datasets 1–4, available as supplementary data at *Bioinformatics Advances* online). The distinction based on the presence or absence of significant differences was made to contrast cases in which paired differences are large and consistent with cases in which they are small or variable.

Furthermore, synthetic data were extended to verify the effectiveness and limitations of the proposed methods with respect to distinguishing true differences from regression to the mean, detecting subgroup heterogeneity, visualizing between-group differences under skewed distributions, and robustness to heteroscedasticity (2 × SD) and outliers (Supplementary Material 1, available as supplementary data at *Bioinformatics Advances* online).

Notably, the trapezoid plot seemed to be robust in distinguishing true differences from regression to the mean, even in the presence of skewed distributions, heteroscedasticity, and outliers. This robustness stems from the simple summary of the ratio of ascending to descending group sizes and the distribution of pairwise slopes. However, subgroup heterogeneity was difficult to detect because the display is limited to two trapezoids; indeed, even for the synthetic data, only the major trend was represented. This may result in a type II error.

The clustered line plot and parallel arrow plot summarized data comprising three intermixed subgroups as clusters in the same direction, aiding the detection of subgroup heterogeneity. Moreover, the parallel arrow plot can display the magnitude and direction of change more intuitively. These plots were also robust in revealing paired differences in the presence of skewed distributions, heteroscedasticity, and outliers (Supplementary Material 1, available as supplementary data at *Bioinformatics Advances* online). However, they tended to aggregate regression to the mean into large clusters, potentially leading to the false detection of a substantial change and a type I error.

These findings suggest that using all three plots together may decrease the respective type I/II errors of each method. For example, even when the trapezoid plot shows approximately symmetrical ascending and descending groups with Q2 lines of equal thickness, the clustered line plot and parallel arrow plot can be consulted to check for the presence of subgroups. Considering that all three methods seemed to be robust in revealing paired differences under skewed distributions, heteroscedasticity, and outliers, they can serve not only as descriptive visualization tools but also as effective screening tools in the early stages of data analysis.

## 4 Case study

**Figure 4:**
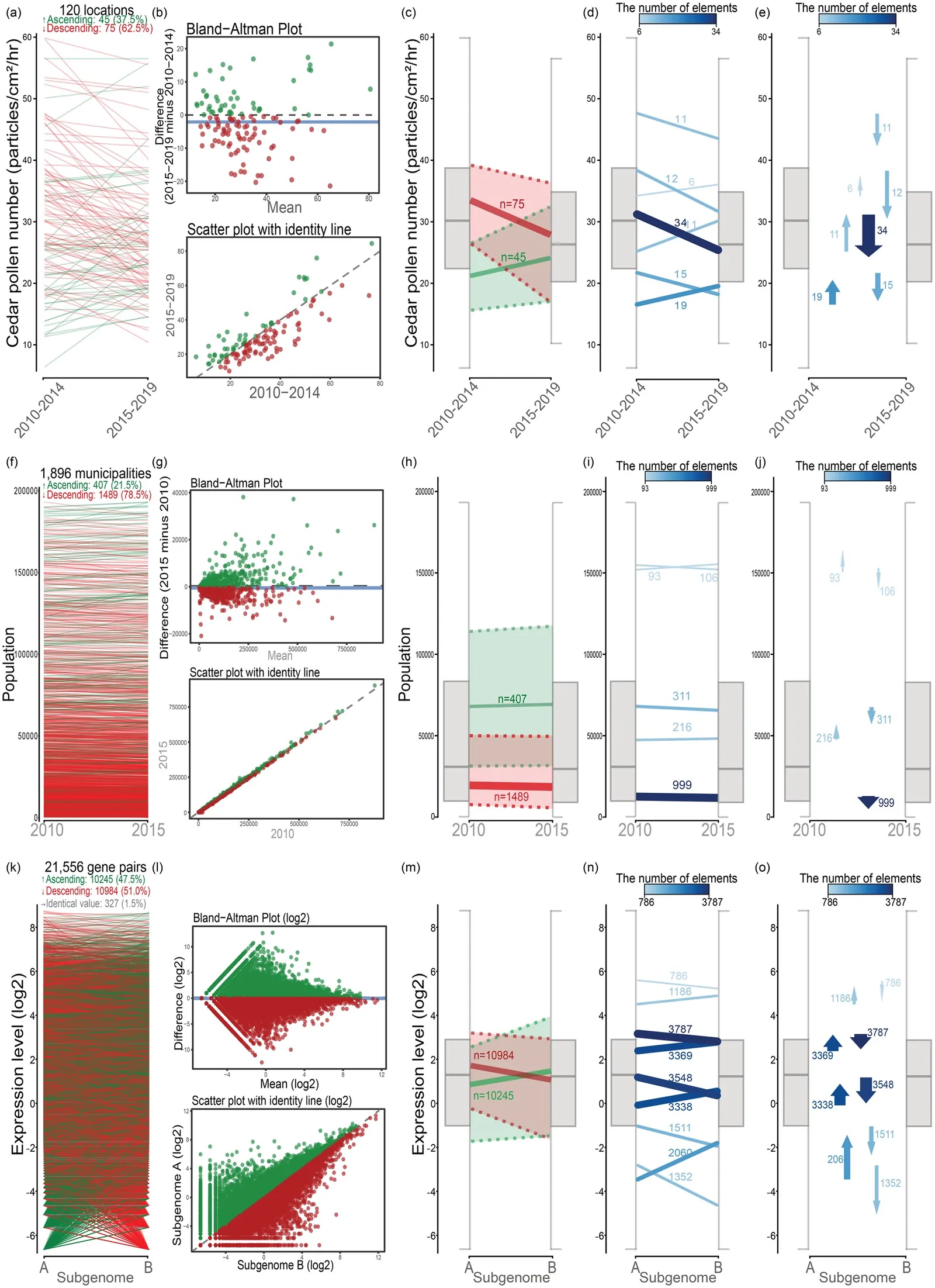
Case studies demonstrating the utility of EZ-Pair Graph. Each row presents one dataset analyzed using five visualization methods. (a, f, k) Slope graph, where upward-sloping lines indicate ascending pairs and downward-sloping lines indicate descending pairs. (b, g, l) Conventional Bland-Altman plot (top) and scatter plot with identity line (bottom). (c, h, m) Trapezoid plot, where solid lines indicate the median (Q2) and dotted lines with shaded bands indicate Q1–Q3 for the ascending (upward-sloping) and descending (downward-sloping) subgroups; band thickness represents density. (d, i, n) Clustered line plot, where each line connects the medians (Q2) of a cluster and line thickness represents cluster size. (e, j, o) Parallel arrow plot, where each arrow connects the medians (Q2) of a cluster and arrow thickness represents cluster size. (a–e) Cedar pollen abundance (2010–2014 vs. 2015–2019, *n* = 120 locations). The trapezoid plot reveals a predominant decreasing trend in pollen counts across locations. (f–j) Japanese population (2010 vs. 2015, *n* = 1896 municipalities). The trapezoid plot reveals divergent demographic trends: populations increased in major cities, but decreased in smaller municipalities. (k–o) Wheat homoeologous gene expression (subgenome A vs. B, *n* = 21 556 pairs, log2-transformed). The slope graph and conventional plots were hindered by visual saturation. The clustered line plot effectively summarizes direction and magnitude, whereas the parallel arrow plot reveals a subtle dominance of subgenome A (51%) over subgenome B (47.5%) .

To further demonstrate the utility of our method, we analyzed cedar pollen data for 120 locations in Japan (2010–2014 vs. 2015–2019; *P *= 2.17 × 10^−3^, Wilcoxon signed-rank test). Conventional visualization methods were used, including the slope graph (Fig. 4a), Bland-Altman plot (Fig. 4b), and scatter plot with identity line (Fig. 4b). For the proposed methods, a trapezoid plot (Fig. 4c), clustered line plot (Fig. 4d), and parallel arrow plot (Fig. 4e) were applied. Although conventional methods revealed a slight overall decreasing trend, clustered line plot and parallel arrow plots (Fig. 4d and e) distinguished decreasing trends at locations with cedar pollen counts that were above the median. This distinction underscores the interpretive advantage of our approach to elucidating trends in paired data.

Hay fever affects a large proportion of the Japanese population and is widely regarded as a national ailment, prompting extensive research efforts (Inomata *et al.* 2022). However, whether the abundance of cedar pollen, which is the primary cause of hay fever, has increased or decreased over time is unclear. This is likely associated with the difficulty in visualizing paired data. Although data for 120 locations exhibited a gradual decreasing trend in most cases, this pattern was not obvious. Therefore, this serves as a clear example of the effectiveness of our approach to visualizing paired data.

We analyzed population data for 1896 Japanese municipalities between 2010 and 2015. Municipalities are the primary data collection unit in the Japanese national census. Japan’s total population decreased by 0.25% during this period, and the Wilcoxon signed-rank test revealed a consistent decreasing trend across municipalities (*P *= 1.50 × 10^−77^). The trapezoid plot (Fig. 4h) reflected divergent trends, with populations increasing in major cities, but decreasing in smaller municipalities. In contrast, local patterns were obscured in other conventional plots (Fig. 4f and g), the clustered line plot (Fig. 4i), and the parallel arrow plot (Fig. 4j). For example, in the Bland-Altman plot (Fig. 4f), the dotted line indicated zero, but the difference from the blue line representing the mean difference was small, making the change difficult to discern. These results highlight the utility of the trapezoid plot for capturing both global and local patterns.

Among gene expression patterns in polyploid wheat (22 395 gene pairs; unexpressed pairs were excluded after log_2_ transformation, resulting in 21 556 gene pairs), 47.5% showed B-subgenome dominance, 51% showed A-subgenome dominance, and 1.5% showed equal dominance (*P *= 2.18 × 10^−7^, Wilcoxon signed-rank test). Conventional visualizations were saturated (Fig. 4k and l), whereas the clustered line plot and the parallel arrow plot (Fig. 4n and o) effectively summarized both direction and magnitude, with color used to reflect density, making the subtle A-subgenome dominance pattern interpretable. In contrast, the trapezoid plot failed to highlight such biased expression patterns because of the nearly balanced number of ascending and descending pairs. The clustered line plot and parallel arrow plot are applicable to other paired comparisons, including expression dominance in F_1_ hybrids (Takahagi *et al.* 2018, Ezoe *et al.* 2024, Zhuo *et al.* 2024).

## 5 Conclusion

Our method for visualizing paired data in three ways (trapezoid plot, clustered line plot, and parallel arrow plot) is useful for effectively summarizing large-scale paired data into a unified axis. The trapezoid plot provides a simply summarized representation of data, but may obscure information when distributions are multimodal or skewed. In contrast, the clustered line plot and parallel arrow plot more faithfully convey such distributional complexity. The proposed method may decrease computational and cognitive burdens associated with large datasets, while revealing patterns that were previously difficult to analyze, thereby making hidden trends more accessible. To improve accessibility, EZ-Pair Graph is also available as a Python package installable via pip (pip install git+https://github.com/010049nn/EZ_pair_graph.git -q), providing both a Python API and a command-line interface. Alternatively, local installation is possible via docker. This enables straightforward integration into existing analysis workflows and Jupyter notebooks.

## Supplementary Material

vbag155_Supplementary_Data

## Data Availability

The data underlying this article are available in (https://github.com/010049nn/EZ_pair_graph). Release archive DOI (10.5281/zenodo.20,437,542).
