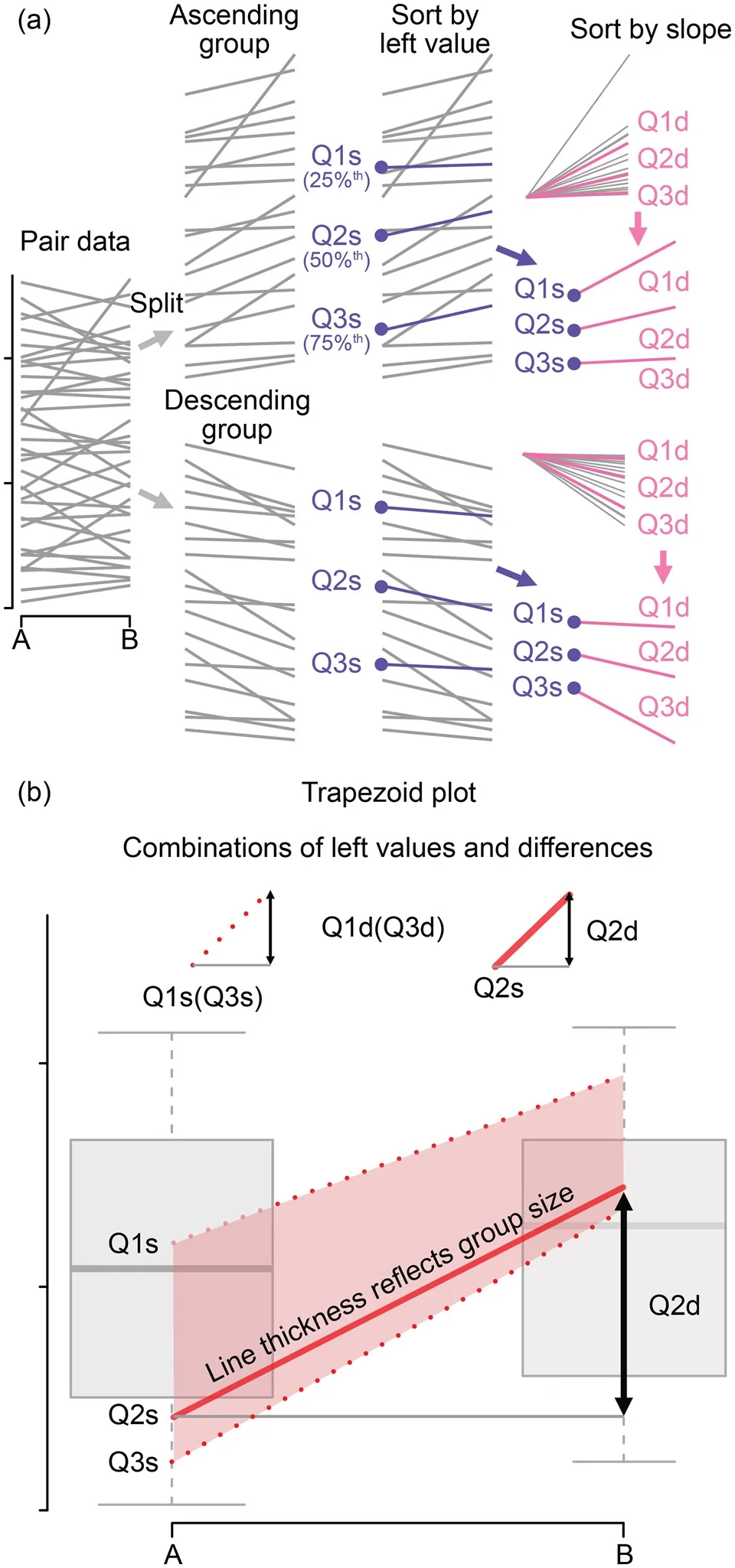# Correction to: EZ-Pair graph: scalable unified-axis visualization method for summarizing large-scale paired data

**DOI:** 10.1093/bioadv/vbag286

**Published:** 2026-10-21

This is a correction to: Akihiro Ezoe, Motoaki Seki, Keiichi Mochida, EZ-Pair graph: scalable unified-axis visualization method for summarizing large-scale paired data, *Bioinformatics Advances*, Volume 6, Issue 1, 2026, vbag155, https://doi.org/10.1093/bioadv/vbag155

In the originally published version of this manuscript, Figures 2 and 3 were inadvertently duplicated.

This error has been corrected so that Figure 2 reads:

**Figure:**