# Supplementary material for: EZ-Pair graph: scalable unified-axis visualization method for summarizing large-scale paired data
Source: Bioinform Adv. 2026 Jun 2;6(1):vbag155. doi: 10.1093/bioadv/vbag155 (PMC13281915; doi:10.1093/bioadv/vbag155)
Supplement: vbag155_Supplementary_Data [file vbag155_supplementary_data.zip › Supplementary_Figure_S1.pdf]

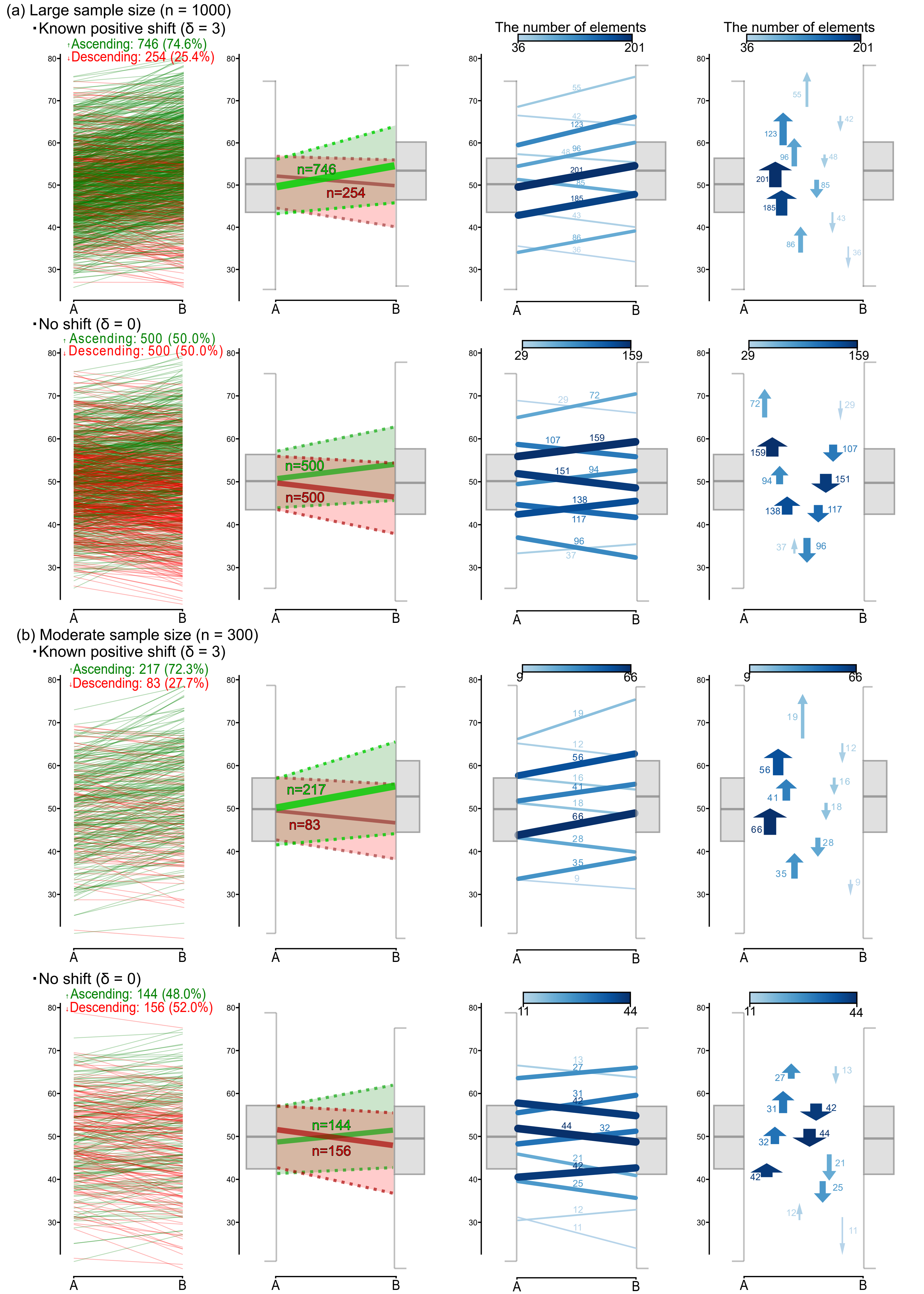

(a) Large sample size (n = 1000)
·Known positive shift (δ = 3)
↑Ascending: 746 (74.6%)
↓Descending: 254 (25.4%)
n=746
n=254
The number of elements
36
201
·No shift (δ = 0)
↑Ascending: 500 (50.0%)
↓Descending: 500 (50.0%)
n=500
n=500
29
159
(b) Moderate sample size (n = 300)
·Known positive shift (δ = 3)
↑Ascending: 217 (72.3%)
↓Descending: 83 (27.7%)
n=217
n=83
9
66
·No shift (δ = 0)
↑Ascending: 144 (48.0%)
↓Descending: 156 (52.0%)
n=144
n=156
11
44
A
B
